# Effect of digital monitoring and counselling on self-management ability in patients with rheumatoid arthritis: a randomised controlled trial

**DOI:** 10.1093/rheumatology/kead709

**Published:** 2023-12-28

**Authors:** Linda C Li, Hui Xie, Lynne M Feehan, Chris Shaw, Na Lu, Smruthi Ramachandran, Ellen Wang, Stephanie Therrien, Julia Mucha, Alison M Hoens, Kelly English, Eileen Davidson, Teresa Liu-Ambrose, Catherine L Backman, John M Esdaile, Kimberly J Miller, Diane Lacaille

**Affiliations:** Department of Physical Therapy, University of British Columbia, Vancouver, BC, Canada; Faculty of Health Sciences, Simon Fraser University, Burnaby, BC, Canada; Department of Physical Therapy, University of British Columbia, Vancouver, BC, Canada; School of Interactive Arts and Technology, Simon Fraser University, Surrey, BC, Canada; Arthritis Research Canada, Vancouver, BC, Canada; Department of Physical Therapy, University of British Columbia, Vancouver, BC, Canada; Department of Physical Therapy, University of British Columbia, Vancouver, BC, Canada; Arthritis Research Canada, Vancouver, BC, Canada; Department of Physical Therapy, University of British Columbia, Vancouver, BC, Canada; Department of Physical Therapy, University of British Columbia, Vancouver, BC, Canada; Arthritis Research Canada, Vancouver, BC, Canada; Arthritis Research Canada, Vancouver, BC, Canada; Department of Physical Therapy, University of British Columbia, Vancouver, BC, Canada; Department of Occupational Science & Occupational Therapy, University of British Columbia, Vancouver, BC, Canada; Arthritis Research Canada, Vancouver, BC, Canada; New Knowledge and Innovation, BC Children’s Hospital and BC Women’s Hospital and Health Centre, Vancouver, BC, Canada; Arthritis Research Canada, Vancouver, BC, Canada

**Keywords:** rheumatoid arthritis, e-health, self-monitoring, physical activity, self-management, patient activation

## Abstract

**Objectives:**

To assess a remote physiotherapist (PT) counselling intervention using self-monitoring tools for improving self-management ability, physical activity participation and health outcomes in people with rheumatoid arthritis (RA).

**Methods:**

Eligible participants were randomly assigned to receive group education, a Fitbit^®^, a self-monitoring app, and PT counselling phone calls (Immediate Group). The Delayed Group received a monthly e-newsletter until week 26, and then the intervention. The primary outcome was Patient Activation Measure (PAM-13). Participants were assessed at baseline, 27 weeks (the primary end point) and 53 weeks. Secondary outcomes included disease activity, pain, fatigue, depression, sitting/walking habits, daily physical activity time and daily awake sedentary time. Generalized Linear Mixed-effect Models (GLMMs) were used to assess the effect of the intervention on the change of each outcome measure from the initiation to 27 weeks after the intervention.

**Results:**

Analysis included 131 participants (91.6% women; 80.2% completed during the COVID-19 pandemic). The mean change of PAM-13 at 27 weeks was 4.6 (Standard Deviation [SD] = 14.7) in the Immediate Group *vs* −1.6 (SD = 12.5) in the Delayed Group. The mean change in Delayed Group at 53 weeks (after the 26-week intervention) was 3.6 (SD = 14.6). Overall, the intervention improved PAM-13 at 27 weeks post-intervention from the GLMM analysis (adjusted coefficient: 5.3; 95% CI: 2.0, 8.7; *P* ≤ 0.001). Favourable intervention effects were also found in disease activity, fatigue, depression and self-reported walking habit.

**Conclusion:**

Remote counselling paired with self-monitoring tools improved self-management ability in people with RA. Findings of secondary outcomes indicate that the intervention had a positive effect on symptom management.

## Graphical abstract

**Figure:**
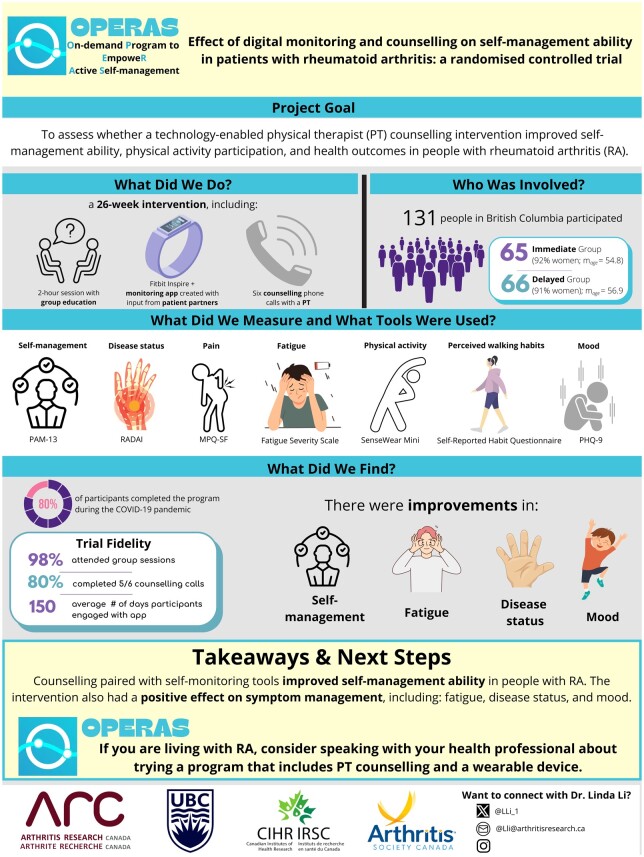

Rheumatology key messages

- Digital self-monitoring tools with remote counselling by a physiotherapist improved self-management ability after 27 weeks.
- This remote intervention also improved rheumatoid arthritis disease activity, fatigue, depression and self-reported walking habit.
- Physiotherapists play a key role in supporting patient self-management regardless of geographic locations.

## Introduction

For people with rheumatoid arthritis (RA), self-management refers to their ability to manage the symptoms and treatments associated with living with a chronic disease [1, 2]. It requires individuals engaging in self-care activities, such as balancing physical activity with rest and sleep [3] and knowing when to seek help from healthcare providers [4]. To achieve better health outcomes, people with RA need to be aware of their symptoms and have the ability and confidence to practice self-care; that is, to be an *activated patient* [5, 6].

Hibbard *et al.* [5] describe *activated patients* as individuals who believe they play a key role in self-managing and are ready to collaborate with their healthcare providers to maintain their wellbeing. Several studies have assessed interventions for enhancing self-management ability—also known as *patient activation* [5]—in people with inflammatory arthritis, but the results were mixed. Mollard and Michaud [7] tested a self-monitoring app for people with RA and found no effect on the Patient Activation Measure (PAM-13) [6]. In a 12-month randomized controlled trial (RCT) of 157 patients with RA, Zuidema *et al.* [8] observed no difference in PAM-13 between those who participated in a 12-month online self-management program and the controls. Similar results were reported by Fortin *et al.* [9] in 541 patients with systemic lupus erythematosus after using a self-paced online self-management program for three months. These findings diverge from the evidence supporting the use of digital tools to enhance participation in self-care activities and improve health outcomes [10]. Interestingly, interventions tailored to the patient’s needs appeared to have an effect on improving patient activation [11–14]. These studies, however, used either a non-randomized design [11] or a short intervention duration [12]. In an RCT of a 6-week tailored education program with a nurse, the initial post-intervention improvement in PAM-13 did not persist at 12 months [13].

The current study assessed the efficacy of a 26-week technology-enabled physiotherapist (PT) counselling intervention aimed at improving the self-management abilities of people with RA (measured by PAM-13). Our hypothesis posited a significant effect of the intervention on the change in PAM-13 from baseline to 27 weeks after its initiation compared with the control participants. Additionally, we explored the effect of the intervention on RA disease activity, pain, fatigue, depression, perceived sitting and walking habits, and physical activity participation.

## Methods

We conducted an RCT with a delay-control design. Participants were randomly assigned to receive the intervention immediately (Immediate Group) or 27 weeks later (Delayed Group). The Delayed Group received a monthly e-newsletter unrelated to RA management during the waiting period. All participants continued their medical treatment and make changes as needed throughout the study. All participants were assessed before randomization at baseline (T0), 27 weeks (T1; primary end point) and 53 weeks (T2) in a natural setting.

Individuals were eligible if they had: (i) a diagnosis of RA confirmed by their rheumatologists in writing; (ii) no surgery or injury to any joints in the past 6 months; (iii) an email address and access to a computer or mobile device; (iv) the ability to speak and understand English; and (v) not previously participated in studies that involved RA symptom monitoring or physical activity tracking and counselling. We excluded people with contraindications to being physically active. Individuals who did not pass the Physical Activity Readiness Questionnaire (PAR-Q) [15] required a physician’s note to determine eligibility.

Participants were recruited from rheumatology clinics in British Columbia, Canada, and through patient groups including Arthritis Consumer Experts and Arthritis Research Canada’s Arthritis Patient Advisory Board. We also posted study information on Facebook, Twitter and Arthritis Research Canada’s website. Ethical approval for this study has been obtained from the University of British Columbia (UBC) Clinical Research Ethics board (H17-03424) and was published in ClinicalTrials.gov (NCT03404245).

### Randomisation and blinding

Randomisation was performed using numbers generated by SAS v9.4 in variable block sizes to ensure allocation concealment by a biostatistician not involved in the study (Eric C. Sayre). After completing baseline measures, participants were enrolled to the Immediate or Delayed Group in 1:1 allocation ratio by a research staff (Johnathan Tam). The study PTs were blinded to the timing of participants randomised to receive the intervention; however, the participants were not blinded. The researcher (L.M.F.) who processed the objectively measured physical activity data and the biostatistician (N.L.) who performed the statistical analysis were blinded to participants’ group assignment.

### Intervention

The 26-week intervention included three components: (i) a 2-h session with group education about physical activity and individual counselling with a PT trained in motivational interviewing [16]; (ii) the use of a wearable device (Fitbit Inspire^TM^) and a monitoring app designed for people with RA; and (iii) six counselling phone calls (15–30 min each) with the PT. Training received by the 11 PTs is detailed in a published study protocol [17]. The counselling followed the Brief Action Planning approach [18] to guide participants in setting physical activity goals, developing an action plan, and identifying barriers and solutions to achieve those goals.

### The app

The *On-demand Program to EmpoweR Active Self-management* (OPERAS) app was co-developed with a multi-media studio (Tactica Interactive, Winnipeg, Canada) and two patient partners (K.E., A.M.H.). A third patient partner (E.D.) provided input to further improve the app design. OPERAS had two components: (i) *Arthritis Health Journal* to monitor disease activity, symptoms and medication use [19]; and (ii) *Activity Tracking* by pairing with a Fitbit^®^ and providing graphics of physical activity levels [20]. Available as a web app and a companion mobile app, OPERAS allowed users to record their disease activity, fatigues, depression, sleep quality, physical activity and treatment use. RA disease activity—recorded with the RAPID-4 [21]—was automatically calculated from the users’ global assessment for pain and global health (both measured with an 11-point visual analogue scale), Multi-dimensional Health Assessment Questionnaire, and the 16-point tender joint count. We chose the RAPID-4 based on the advice of patient partners and the experience of clinicians in the research team that the 16-joint count was useful during the counselling sessions. Time spent in physical activity and sleep was recorded by Fitbit^®^ and synced to the app. Users could see their disease activity scores, symptom ratings and physical activity on a dashboard and share this information through a secure portal with their healthcare providers.

### Technology-enabled physiotherapist counselling

Participants recorded their symptoms on the app at least twice a week during periods of active disease (i.e. a disease activity score above 4), and once every 2 weeks during periods of stable disease (i.e. no sudden increases in disease activity score). Individuals who did not record their disease activity for >2 weeks received an email reminder. A PT called the participant at weeks 2, 4, 6, 8, 13 and 26 to review symptoms, treatment use, physical activity participation and self-care goals. Participants were counselled to modify their goals as they wished. After the intervention period, participants could keep their Fitbit^®^ and app account, but did not have access to the PT.

### Primary outcome

Self-management ability was assessed with PAM-13, which is a measure of an individual’s perceived knowledge, skills and confidence in managing chronic disease [5, 6]. The aggregate raw score was converted to a scale of 0–100. Hibbard [5] described a 4- level activation model based on the standardized scores of PAM-13: Level 1: believing an active role is important (score 0–47.0); Level 2: having confidence and knowledge to take action (47.1–55.1); Level 3: taking action (55.2–67.0); and Level 4: maintaining healthy behaviors despite setbacks (67.1–100). A change of 3–4 points for an individual is associated with a difference between engaging and not engaging in a self-care behaviour [22].

### Secondary outcomes

We assessed disease status with the Rheumatoid Arthritis Disease Activity Index (RADAI) [23]. Pain was measured with the McGill Pain Questionnaire (MPQ-SF) [24]. We measured fatigue using the Fatigue Severity Scale, a 9-item questionnaire [25]. An improvement of 6.6% is considered clinically important in this population [26, 27]. Participants’ mood was assessed using the Patient Health Questionnaire-9 (PHQ-9) which consisted of nine questions that correspond to the diagnostic criteria for major depressive disorder [28]. A total score of >11 indicates the presence of major depressive disorder [29].

The Self-Reported Habit Index [30, 31] was used to assess characteristics of habitual behaviour, such as sitting during leisure time at home, sitting during usual occupational activities, and walking outside for ≥10 min. It is a 12-item scale, with higher scores indicating a stronger habit or behaviour that is done frequently, automatically, and done without thinking about it.

We measured participants’ physical activity and awake sedentary time with a SenseWear Mini, a research-grade multi-sensor device, using the average from 4–6 days with at least 20 h of wear [32, 33]. Participants wore the SenseWear over the triceps of their non-dominant arm for 7 days. We calculated: (i) average time in bouts of moderate/vigorous physical activity (MVPA) per day [a bout was defined as ≥10 consecutive min at the level of ≥3 MET (Metabolic Equivalent of Task), with allowance for interruption of ≤2 min below the threshold [34]]; and (ii) average daily awake sedentary time, with an energy expenditure of ≤1.5 MET, occurring in bouts of ≥20 min during waking hours [35, 36].

### Trial fidelity, changes due to COVID-19 and adverse events

Intervention fidelity was measured by participants’ attendance in the group education and PT counselling sessions, and their engagement with the self-monitoring app and Fitbit^®^. Since the participants’ Fitbit^®^ data was synchronised automatically when they logged into the app, we measured engagement during the intervention period by: (i) the duration of use, defined as the difference between the first and last day they added a record in the app; (ii) the average number of days they added a record in the app; and (iii) the average number of days between two record entries.

The COVID-19 pandemic had no impact on the trial design, intervention and outcomes, but caused a change to the intervention delivery and data collection [37]. Originally, the initial 2-h session was delivered in person. Due to pandemic restrictions, sessions after March 2020 were delivered via Zoom. Between March and July 2020, we halted data collection with the SenseWear due to the university’s infectious disease protocol. Individuals could decline to wear the SenseWear even after the protocol was eased. Participants reported all serious adverse events, including falls, cardiovascular and musculoskeletal events [38], to the research coordinator during the study. They also recorded adverse events related to physical activity in the follow-up questionnaires at weeks 27 and 53.

### Data analysis

We followed a predefined plan [17] and performed an intention-to-treat analysis using SAS software, version 9.4. Generalized Linear Mixed-effect Models (GLMMs), adjusting for sex as a covariate, were conducted to investigate the primary and secondary objectives [17]. These models included all participants and accounted for data missing at random without the need to perform explicit imputations of the missing values [39]. The dependent variables in these models were the longitudinal outcomes measured at the three measurement time points. These models contained individual-specific random intercepts and time slope effects, and fixed effects that assign a separate outcome mean parameter for each combination of time point by group (Immediate *vs* Delayed). We assessed the effect of the intervention on the change of each outcome measure (from the intervention initiation to 27 weeks) by combining the following two contrasts using the GLMM: (i) the between-group difference of the Delayed and Immediate Groups in the outcome change: T1-T0; and (ii) the within-group difference before and after the intervention for the Delayed Group: T2-T1 *vs* T1-T0. We used the sandwich estimators for GLMM [40] to compute empirical standard errors that were robust to model specifications and distributional assumptions. An unstructured variance–covariance matrix was used to model the within-participant error variance–covariance for continuous outcomes.

### Sample size calculation

Turner *et al.* [41] reported that patients with chronic diseases who completed a self-management program improved their mean PAM-13 scores from 52.2–60.2 (standardized effect size = 0.65). Based on a difference of 8 points, an estimated standard deviation (SD) of 12.4, and a 2-tailed analysis of covariance (ANCOVA), 102 participants (51 per group) were needed (90% power; α-level 0.05). We planned to recruit a total sample size of 134, with 67 participants in each group, to allow for a 24% attrition rate.

## Results

**Figure 1.**
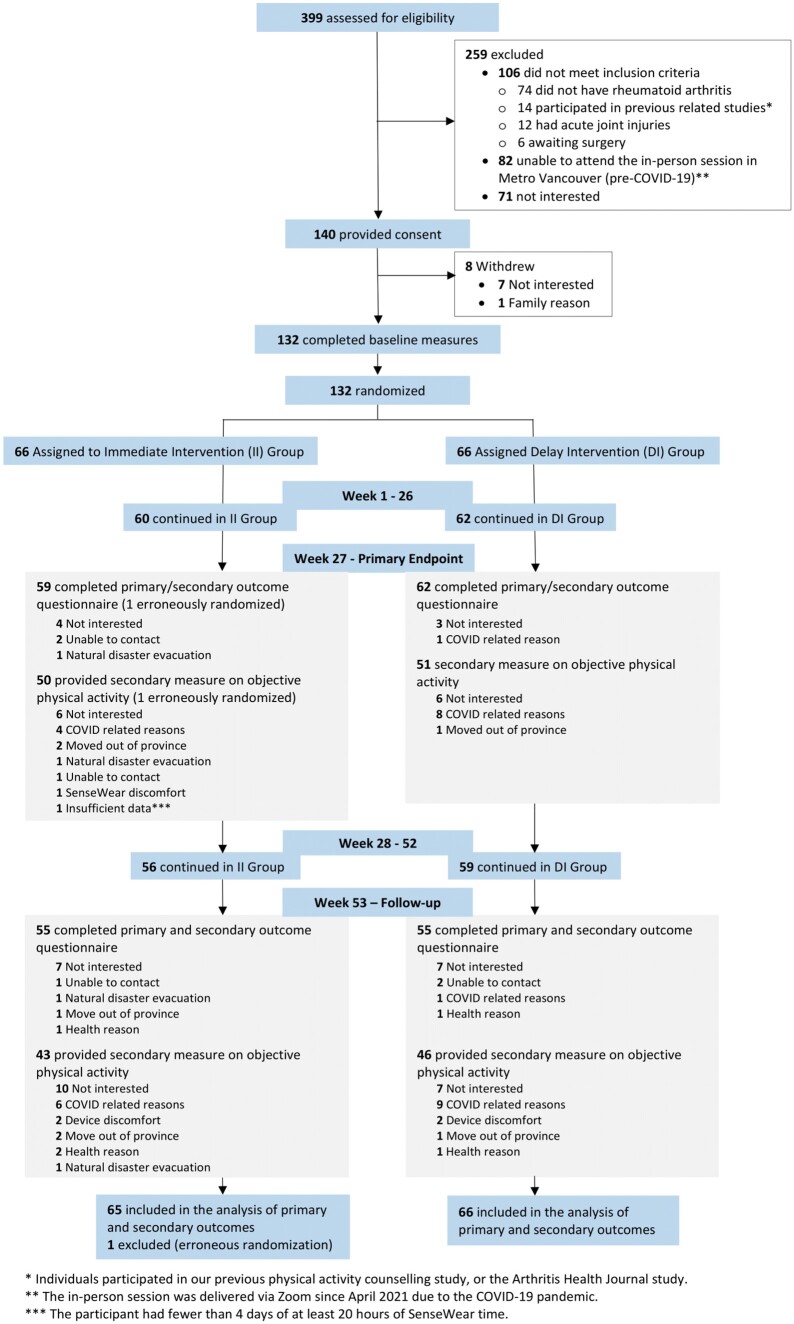
Consolidated Standards of Reporting Trials (CONSORT) flowchart

From 2019 to 2022, 399 people expressed interest, 140 consented and 132 were enrolled (Fig. 1). One individual in the Immediate Group revealed that he did not have RA after randomization; hence, 131 were included in the analysis (Immediate: 65, 92% women; Delayed: 66, 91% women). Groups were similar in age (Immediate: 54.8 [SD 13.1] years; Delayed: 56.9 [SD 13.2] years). A total of 80.2% (*n* = 105) participated in the study during the COVID-19 pandemic (Table 1). Of the 131 participants, 120 (92%) and 110 (84%) completed the online self-reported measures, while 100 (76%) and 89 (68%) completed the physical activity measures with SenseWear at weeks 27 and 53, respectively (Fig. 1).

**Table 1.** Baseline characteristics of participants

	All	Immediate Group	Delayed Group
	(N = 131)	(N = 65)	(N = 66)
Women; *n* (%)	120 (91.6)	60 (92.3)	60 (90.9)
Age; years (SD)	55.8 (13.1)	54.8 (13.1)	56.9 (13.2)
Marital status; *n* (%)			
Married/Common law	88 (67.2)	44 (67.7)	44 (66.7)
Separated/Divorced	23 (17.6)	14 (21.5)	9 (13.6)
Widowed/Never married	20 (15.3)	7 (10.8)	13 (19.7)
University degree or trades certificate; *n* (%)	61 (46.6)	32 (49.2)	29 (43.9)
Gross annual household income in Canadian dollars; *n* (%)			
$40 000 or under	32 (16.8)	11 (16.9)	11 (16.7)
$40 001–$80 000	28 (21.4)	12 (18.5)	16 (24.2)
Over $80 000	62 (47.3)	37 (56.9)	25 (37.9)
No answer	19 (14.5)	5 (7.7)	14 (21.2)
Disease duration,^a^ years (SD)	10.5 (11.0)	9.2 (10.9)	11.7 (11.2)
Body mass index; kg/m^2^ (SD)	26.9 (6.4)	27.7 (6.8)	26.0 (6.0)
Experience with health apps; *n* (%)			
<1 year	73 (55.7)	37 (56.9)	36 (54.6)
1–3 years	23 (17.5)	10 (15.4)	13 (19.7)
≥4 years	20 (15.3)	11 (16.9)	9 (13.6)
No answer	15 (11.5)	7 (10.8)	8 (12.1)
Level of patient activation;^c^*n* (%)			
1 (score <47)	11 (8.4)	7 (10.8)	4 (6.1)
2 (47.1–55.1)	15 (11.4)	7 (10.8)	8 (12.1)
3 (55.2–67)	42 (32.1)	25 (38.4)	17 (25.8)
4 (>67.1–100)	63 (48.1)	26 (40.0)	37 (56.0)
Completed the study during the COVID-19 pandemic^b^	105 (80.2)	51 (78.5)	54 (81.8)

a Year of diagnosis was available in 33/66 of the Immediate Group and 35/66 of the Delayed Group.

b Defined as completed 6-month (T1) assessment after 11 March 2020 when the World Health Organization declared the COVID-19 a pandemic.

c Assessed by the Patient Activation Measure.

MVPA: moderate/vigorous physical activity; SD: standard deviation.

**Figure 2.**
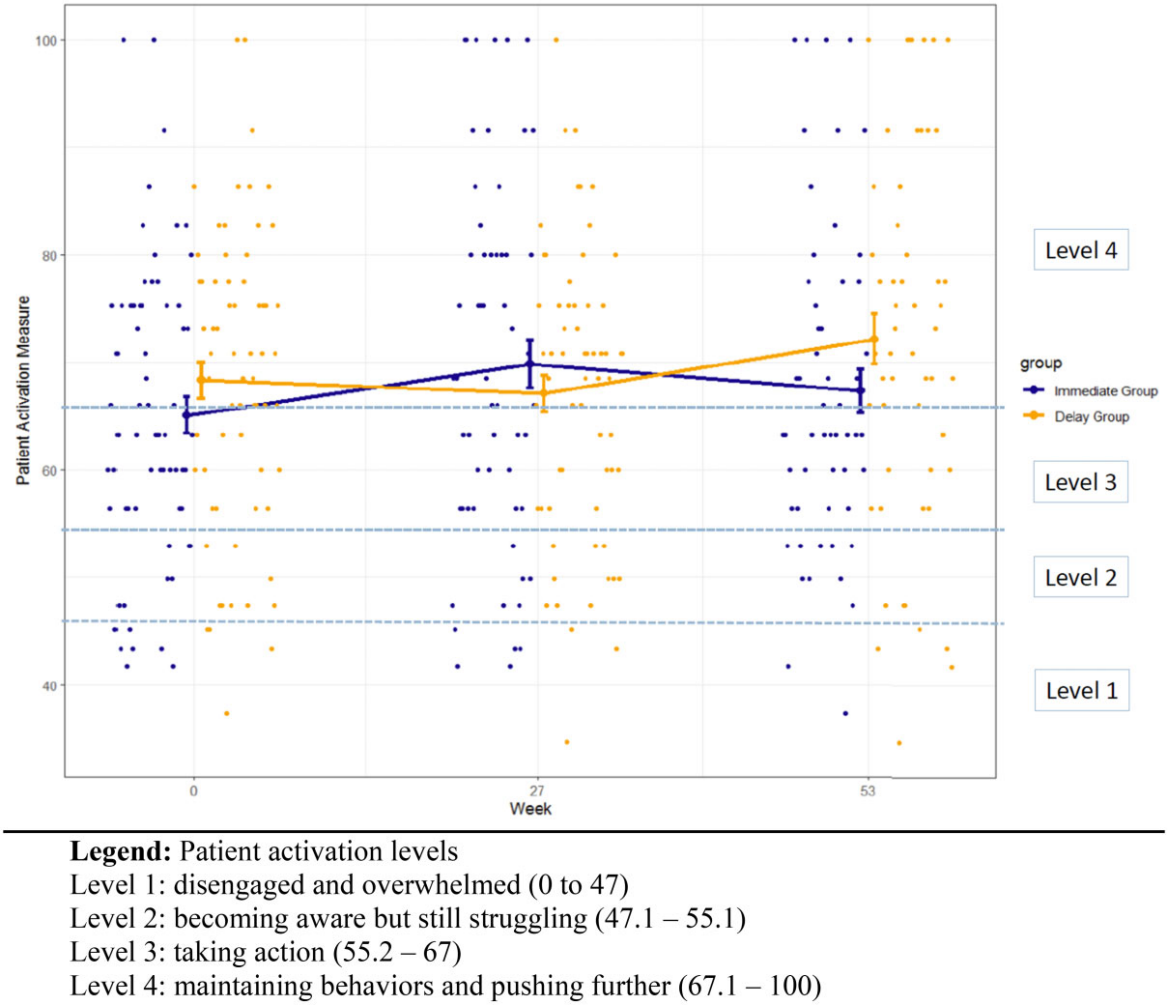
Changes in Patient Activation Measure in both groups across time

The baseline mean PAM-13 scores were 65.1 (SD 13.7) for the Immediate Group and 68.3 (SD 13.9) for the Delayed Group (Table 2). Most participants (Immediate: 78%; Delayed: 82%) were at the Patient Activation Level 3 or 4 (Table 1). The mean change of PAM-13 from T0 to T1 was 4.6 (SD = 14.7) in the Immediate Group *vs* −1.6 (SD = 12.5) in the Delayed Group. The mean change in the Delayed Group from T1 to T2 (after the 26-week intervention) was 3.6 (SD = 14.6). Fig. 2 shows changes in PAM-13 for both groups across time. Twenty-six participants in the Immediate Group *vs* 18 in the Delayed Group reported ≥4-point improvement on PAM-13 at T1 [22].

**Table 2.** Participant outcomes

	Immediate Group (SD^b^)			Delayed Group (SD)			
	Baseline (T0)	27 weeks (T1)	53 weeks (T2)	Baseline (T0)	27 weeks (T1)	53 weeks (T2)	Unadjusted between group difference for T1-T0 (95% CI^c^)
	*n = 65*	*n = 58*	*n = 55*	*n = 66*	*n = 62*	*n = 55*	

Patient Activation Measure (0–100; higher = better)	65.1 (13.7)	69.8 (17.2)	67.3 (15.1)	68.3 (13.9)	67.1 (13.5)	72.2 (17.5)	6.2 (1.3, 11.1)^a^
Rheumatoid Arthritis Disease Activity Index (0–10; lower = better)	3.6 (1.9)	3.0 (2.0)	2.8 (2.0)	3.7 (1.9)	3.6 (1.8)	2.8 (1.8)	−0.4 (−1.0, 0.2)
McGill Pain Questionnaire (0–45; lower = better)	11.0 (8.2)	9.1 (8.0)	9.2 (7.9)	10.2 (7.5)	10.0 (7.1)	9.5 (8.5)	−1.1 (−3.2, 1.0)
Fatigue Severity Scale (1–7; lower = better)	4.7 (1.4)	4.3 (1.5)	4.5 (1.5)	4.6 (1.3)	4.6 (1.3)	4.4 (1.5)	−0.3 (−0.7, -0.0)^a^
Patient Health Questionnaire 9 (0–27; lower = better)	7.6 (5.6)	5.0 (3.8)	4.8 (4.0)	7.0 (4.9)	6.3 (5.3)	5.4 (5.0)	−1.8 (−3.3, -0.2)^a^
Self-Reported Habit Index (1–7; higher = stronger habit)							
Sitting at Work subscale	4.6 (1.4)	4.6 (1.4)	4.5 (1.6)	4.7 (1.7)	4.6 (1.6)	4.6 (1.8)	0.0 (−0.5, 0.5)
Sitting at Leisure subscale	4.5 (1.4)	4.5 (1.1)	4.4 (1.3)	4.6 (1.3)	4.7 (1.5)	4.6 (1.6)	−0.2 (−0.6, 0.2)
Walking subscale	4.5 (1.6)	4.6 (1.7)	4.8 (1.6)	4.5 (1.8)	4.3 (1.9)	4.7 (1.7)	0.3 (−0.1, 0.8)

	*n = 58*	*n = 49*	*n = 43*	*n = 60*	*n = 51*	*n = 46*	

Daily MVPA time^d^ [min]	36.8 (35.9)	42.0 (41.5)	42.4 (44.0)	38.4 (38.6)	45.5 (54.5)	47.8 (55.8)	2.5 (−10.1, 15.1)
Daily awake sedentary time^e^ [min]	514.9 (176.1)	539.8 (186.9)	546.7 (163.2)	467.8 (175.2)	504.2 (184.4)	504.0 (164.2)	−57.5 (−112.9, -2.0)^a^

a *P < *0.05.

b SD = standard deviation.

c CI = confidence interval.

d Daily MVPA (moderate/vigorous physical activity) time was defined as ≥3 MET and in bouts ≥10 min with allowance for 2-min interruptions.

e Daily awake sedentary time was defined as ≤1.5 MET in bouts ≥20 min.

### Effects of the intervention on patient activation

Table 3 presents the results of the intervention effect on the change of PAM-13 from intervention initiation to 27 weeks post-intervention using the GLMM. A statistically significant result was observed in the intervention effect (adjusted coefficient: 5.3; 95% CI: 2.0, 8.7; *P* ≤ 0.001). The effect was underpinned by both the between-group difference (adjusted coefficient: 5.4; 95% CI: 0.8, 9.9), and the within-group change in the Delayed Group from the no-intervention to intervention period (adjusted coefficient: 5.2; 95% CI: 1.1, 9.2). No change was found in the sensitivity analysis including the erroneously randomized individual (Supplementary Tables S1 and S2, available at *Rheumatology* online).

**Table 3.** Intervention effect estimates using general linear mixed-effects models

	Adjusted Group effect Immediate vs. Delayed Coefficient (95% CI)			
	Contrast 1^a^	Contrast 2^a^	Contrast 3^a^ (Intervention effect)	Cohen’s d
Patient Activation Measure	5.4 (0.8, 9.9)^b^	5.2 (1.1, 9.2)^b^	5.3 (2.0, 8.7)^b^	0.39
Rheumatoid Arthritis Disease Activity Index	−0.5 (−1.0, 0.1)	−0.8 (−1.3, −0.2)^b^	−0.6 (−1.1, −0.2)^b^	0.36
McGill Pain Questionnaire	−1.1 (−3.0, 0.7)	−0.4 (−2.0, 1.1)	−0.8 (−2.1, 0.6)	0.14
Fatigue Severity Scale	−0.3 (−0.7, −0.2)^b^	−0.3 (−0.5, 0.0)^b^	−0.3 (−0.5, −0.1)^b^	0.32
Patient Health Questionnaire-9	−1.6 (−2.9, −0.3)^b^	−0.8 (−1.9, 0.3)	−1.3 (−2.3, −0.3)^b^	0.30
Self-Reported Habit Index				
Sitting at Work subscale	0.0 (−0.4, 0.4)	0.0 (−0.4, 0.3)	0.0 (−0.3, 0.3)	0
Sitting at Leisure subscale	−0.2 (−0.6, 0.1)	−0.1 (−0.5, 0.2)	−0.2 (−0.5, 0.1)	0.17
Walking subscale	0.3 (−0.1, 0.8)	0.4 (0.0, 0.8)^b^	0.4 (0.0, 0.7)^b^	0.30
Daily MVPA time^c^	2.0 (−10.1, 14.1)	6.0 (−7.3, 19.4)	4.0 (−4.6, 12.7)	0.13
Daily awake sedentary time^d^	−37.9 (−92.6, 16.8)	2.8 (−30.0, 35.6)	−13.3 (−46.7, 20.1)	0.1

a **Contrast 1**: Immediate Group T1–T0 vs Delayed Group T1–T0.

**Contrast 2**: Delayed Group T2–T1 *vs* Delayed Group T1–T0.

**Contrast 3**: Average of Contrast 1 and Contrast 2.

b *P* *<* 0.05.

c Daily MVPA (moderate/vigorous physical activity) time was defined as ≥3 MET and in bouts ≥10 min with allowance for 2-min interruptions.

d Daily awake sedentary time was defined as ≤1.5 MET in bouts ≥20 min.

### Effects of the intervention on secondary outcomes

We found intervention effects in the RADAI (adjusted coefficient: −0.6, 95% CI: −1.1, −0.2; *P* = 0.005), Fatigue Severity Scale (adjusted coefficient: −0.3, 95% CI: −0.5, −0.1; *P* = 0.01), PHQ-9 (adjusted coefficient: −1.3, 95% CI: −2.3, −0.3; *P* = 0.01), and Walking subscale of the Self-Reported Habit Index (adjusted coefficient: 0.4, 95% CI: 0.0, 0.7; *P* = 0.04). No statistically significant effect was observed in other self-reported outcomes or objectively measured physical activity outcomes. (Table 3; Supplementary Fig. S1, available at *Rheumatology* online).

### Intervention fidelity and adverse events

In the Immediate Group, one participant dropped out and never received the intervention. Of the remaining 64 participants, all attended the education and counselling sessions, and 52 completed at least five of the six counselling phone calls. During the intervention period, participants used the app for an average of 150.1 days (SD 44.5) and recorded information on the app 22.8 times (SD 26.9). The mean duration between two records was 12.1 days (SD 9.4). Participants appeared to continue using the app for tracking their health in weeks 27–52 after the counselling calls ended (Table 4).

**Table 4.** Intervention fidelity

Adherence criterion	Intervention period^a^ (183 days)		Study period (365 days)
	Immediate Group (*n* = 65)	Delayed Group (*n* = 66)	Immediate Group (*n* = 65)
1. Attended group education and met with a physiotherapist at the initial counselling session (%)	64 (98.5)	59 (89.3)	—
2. Completed ≥5 of 6 counselling phone calls with the physiotherapist (%)	52 (80.0)	41 (62.1)	—
3. Use of the OPERAS app^a^			
Number of times logged in^b^ (SD)	22.8 (26.9)	16.2 (11.0)	32.9 (42.4)
Days from the first to last log-in^c^ (SD)	150.1 (44.5)	144.8 (51.0)	245.9 (113.4)
Days between log-ins^c^ (SD)	12.1 (9.4)	12.3 (7.4)	13.4 (9.4)

a Immediate Group received the intervention in weeks 1–26. Participants kept their OPERAS app account and Fitbit during weeks 27–52 but had no access to a physiotherapist. Delayed Group received the intervention in weeks 27–52.

b Only participants who initiated the app were included: Immediate Group = 62; Delayed Group = 58.

c Only participants who logged in the app on at least two different days were included: Immediate Group = 62; Delayed Group = 56.

After starting the intervention, one participant from the Immediate Group reported muscle pain due to an increase in physical activity after starting the intervention. Falls, unrelated to the intervention, were reported by two participants from each group. No other adverse events attributable to the intervention were reported.

## Discussion

In this RCT, we found that PT counselling with the use of self-monitoring tools improved self-management ability in people with RA. Although there is no established minimal important difference for PAM-13 at a group level, a 3-to-4-point improvement is considered clinically important for individuals [22]. A total of 40% in the Immediate Group and 27% in the Delayed Group achieved this benchmark at 27 weeks. We also observed intervention effects on the participants’ RA disease activity, fatigue, depression and perceived walking habit.

Participants in this study had a baseline mean score of PAM-13 of 66.7 (Level 3), meaning they were ready to ‘take action’ in self-management. While we observed an effect on PAM-13 after the 27-week intervention in the Immediate Group, the improvement did not sustain at follow-up. Our results were similar to those of Grønning *et al.* [13] who found that the improvement on PAM-13 from a 6-week tailored education program with a nurse did not persist at 12 months. Of note, participants in our study kept their Fitbit and monitoring app after the intervention period. The engagement with the digital components was good with participants interacting with the app approximately every two weeks. It is possible that periodic booster sessions of physiotherapist counselling may be needed to sustain the improvement in individuals’ RA self-management abilities. This hypothesis can be tested in future studies.

Our intervention integrated subjectively reported data and objective data from a Fitbit to support participants’ self-care practices. Notably, most previous studies of self-monitoring interventions tracked disease-related parameters or physical activity, but not both [42–44]. Guided by our patient partners, the self-monitoring app was intentionally designed to visually display individuals’ symptoms, disease activity and physical activity participation over time on a dashboard. This app offered rich information to guide the PT counselling. This has contributed to improving self-management abilities compared with the controls, despite the previous studies of digital interventions reporting negative results [7–9].

Unlike previous studies using monitoring tools with or without counselling [42, 44–47], the current intervention had no effect on participants’ physical activity time and awake sedentary time. A systematic review by Brickwood *et al.* [42] reported that multi-faceted interventions, including the use of wearables and counselling, had a moderate effect on improving physical activity in people with chronic diseases. In a meta-analysis of 75 studies, Vetrovsky and colleagues [44] compared adding other components to self-monitoring devices with using the devices alone in promoting physical activity. They found that combining other components with self-monitoring provided an additional benefit of about 900 steps/day compared with self-monitoring alone. Among the additional components used, only phone or video counselling provided additional benefits. While these components were employed in our intervention, most participants were involved in the study during the COVID-19 pandemic. As revealed in our post-hoc interviews [48], some participants changed their usual routines due to the pandemic restrictions. While some were unable to access their exercise classes, others found new ways to practice self-care while working from home. The change in opportunities for participants to engage in physical activities during the pandemic might have been a stronger driver for individuals to participate in physical activity than the intervention itself. This hypothesis could be further explored by examining physical activity intervention studies conducted before and during the pandemic.

This study has several limitations. First, we did not collect information on treatment changes during the study period. Hence, it is unclear if the intervention influenced participants’ help-seeking behaviours and treatment use for RA. Second, our baseline PAM-13 scores indicated that individuals were already activated when they enrolled. Therefore, the results may not be generalizable to people with RA who were at a lower level of activation and had different needs for self-management support. Third, 92% of participants identified as women; the results may not be generalizable to individuals of other gender identities. Finally, information on participants’ race or ethnicity were not collected; hence, we are unable to comment on the generalizability of this intervention for people of different backgrounds.

## Conclusions

This study demonstrated that remote PT counselling paired with the use of self-monitoring tools improved self-management ability of people with RA. The favourable results on disease activity, fatigue, depression and perceived walking habit indicate that the intervention has a positive effect on RA management. Our results offer evidence in support of using this intervention to enhance individuals’ ability to engage in self-care activities. Further research on the cost-effectiveness of the intervention will facilitate its integration in the care for patients with RA.

## Supplementary material

Supplementary material is available at *Rheumatology* online.

## Supplementary Material

kead709_Supplementary_Data

## Data Availability

The datasets generated and analysed during the current study are available from the corresponding author on reasonable request.
