# Supplementary material for: Effect of digital monitoring and counselling on self-management ability in patients with rheumatoid arthritis: a randomised controlled trial
Source: Rheumatology (Oxford). 2023 Dec 28;64(1):310–20. doi: 10.1093/rheumatology/kead709 (PMC11701315; doi:10.1093/rheumatology/kead709)
Supplement: kead709_Supplementary_Data [file kead709_supplementary_data.docx]

# Supplementary Table S1: Sensitivity analysis - participant outcomes including the erroneously randomized individual

|  | Immediate Group (SD^‡^) | | | Delayed Group (SD) | | |  |
| --- | --- | --- | --- | --- | --- | --- | --- |
|  | **Baseline**  **(T0)** | **27 weeks**  **(T1)** | **53 weeks**  **(T2)** | **Baseline**  **(T0)** | **27 weeks**  **(T1)** | **53 weeks**  **(T2)** | **Unadjusted between group difference for T1-T0 (95% CI^‡‡^)** |
|  | ***n = 65*** | ***n = 58*** | ***n = 55*** | ***n = 66*** | ***n = 62*** | ***n = 55*** |  |
| Patient Activation Measure (0-100; higher = better) | 65.1 (13.6) | 69.9 (17.0) | 67.3 (15.1) | 68.3 (13.9) | 67.1 (13.5) | 72.2 (17.5) | 6.2 (1.4, 11.1) |
| Rheumatoid Arthritis Disease Activity Index (0-10; lower = better) | 3.6 (1.9) | 3.0 (2.0) | 2.8 (2.0) | 3.7 (1.9) | 3.6 (1.8) | 2.8 (1.8) | -0.4 (-1.0, 0.2) |
| McGill Pain Questionnaire (0-45; lower = better) | 10.9 (8.3) | 9.3 (8.1) | 9.2 (7.9) | 10.2 (7.5) | 10.0 (7.1) | 9.5 (8.5) | -0.8 (-2.9, 1.4) |
| Fatigue Severity Scale (1-7; lower = better) | 4.6 (1.4) | 4.3 (1.5) | 4.5 (1.5) | 4.6 (1.3) | 4.6 (1.3) | 4.4 (1.5) | -0.3 (-0.7, -0.0) |
| Patient Health Questionnaire-9 (0-27; lower = better) | 7.6 (5.6) | 5.0 (3.8) | 4.8 (4.0) | 7.0 (4.9) | 6.3 (5.3) | 5.4 (5.0) | -1.7 (-3.3, -0.2) |
| Self-Reported Habit Index (1-7; higher = stronger habit) | | | |  |  |  |  |
| Sitting at Work subscale | 4.6 (1.4) | 4.6 (1.4) | 4.5 (1.6) | 4.7 (1.7) | 4.6 (1.6) | 4.6 (1.8) | 0.0 (-0.5, 0.5) |
| Sitting at Leisure subscale | 4.5 (1.3) | 4.5 (1.1) | 4.4 (1.3) | 4.6 (1.3) | 4.7 (1.5) | 4.6 (1.6) | -0.2 (-0.6, 0.2) |
| Walking subscale | 4.5 (1.6) | 4.6 (1.7) | 4.8 (1.6) | 4.5 (1.8) | 4.3 (1.9) | 4.7 (1.7) | 0.3 (-0.1, 0.8) |
|  | ***n = 58*** | ***n = 49*** | ***n = 43*** | ***n = 60*** | ***n = 51*** | ***n = 46*** |  |
| Daily MVPA time^†^ [mins] | 36.8 (35.6) | 42.0 (41.0) | 42.4 (44.0) | 38.4 (38.6) | 45.5 (54.5) | 47.8 (55.8) | 2.7 (-9.8, 15.1) |
| Daily awake sedentary time^††^ [mins] | 514.5 (174.6) | 538.9 (185.1) | 546.7 (163.2) | 467.8 (175.2) | 504.2 (184.4) | 504.0 (164.2) | -57.8 (-112.6, -3.0) |

^*^ SD = Standard deviation

^†^ Daily MVPA (moderate/vigorous physical activity) time was defined as >3 MET and in bouts >10 minutes with allowance for 2-minute interruptions

^††^ Daily awake sedentary time was defined as <1.5 MET in bouts >20 minutes

# Supplementary Table S2: Sensitivity analysis - Results of general linear mixed-effects models including the erroneously randomized individual

|  | Adjusted Group effect Immediate vs. Delayed Coefficient (95% CI) | | |
| --- | --- | --- | --- |
|  | **Contrast 1^‡^** | **Contrast 2^‡^** | **Contrast 3^‡^**  **(Intervention effect)** |
| Patient Activation Measure | 5.4 (0.9, 9.9)* | 5.1 (1.1, 9.2)* | 5.3 (2.0, 8.7)* |
| Rheumatoid Arthritis Disease Activity Index | -0.5 (-1.0, 0.0) | -0.8 (-1.3, -0.2)* | -0.6 (-1.1, -0.2)* |
| McGill Pain Questionnaire | -0.8 (-2.8, 1.1) | -0.4 (-2.0, 1.1) | -0.8 (-2.1, 0.6) |
| Fatigue Severity Scale | -0.3 (-0.7, -0.0)* | -0.3 (-0.5, 0.0)* | -0.3 (-0.5, -0.1)* |
| Patient Health Questionnaire-9 | -1.6 (-2.9, -0.3)* | -0.8 (-1.9, 0.4) | -1.3 (-2.3, -0.3)* |
| Self-Reported Habit Index |  |  |  |
| Sitting at Work subscale | 0.0 (-0.4, 0.4) | 0.0 (-0.4, 0.3) | 0.0 (-0.3, 0.3) |
| Sitting at Leisure subscale | -0.2 (-0.6, 0.1) | -0.1 (-0.5, 0.2) | -0.2 (-0.5, 0.1) |
| Walking subscale | 0.3 (-0.1, 0.8) | 0.4 (0.1, 0.8)* | 0.4 (0.0, 0.7)* |
| Daily MVPA time ^†^ | 2.2 (-9.8, 14.1) | 6.1 (-7.3, 19.4) | 4.0 (-4.6, 12.7) |
| Daily awake sedentary time^††^ | -38.4 (-92.5, 15.7) | 2.8 (-30.0, 35.6) | -13.1 (-46.7, 20.1) |

**^‡^** **Contrast 1**: Immediate Group T0–T1 vs. Delayed Group T0–T1

**Contrast 2**: Delayed Group T1–T2 vs. Delayed Group T0–T1

**Contrast 3**: Average of Contrast 1 and Contrast 2

* *P* < 0.05

^†^ Daily MVPA (moderate/vigorous physical activity) time was defined as >3 MET and in bouts >10 minutes with allowance for 2-minute interruptions

^††^Daily awake sedentary time was defined as <1.5 MET in bouts >20 minutes

# Supplementary Figure S1: Changes in secondary outcome measures in both groups across time


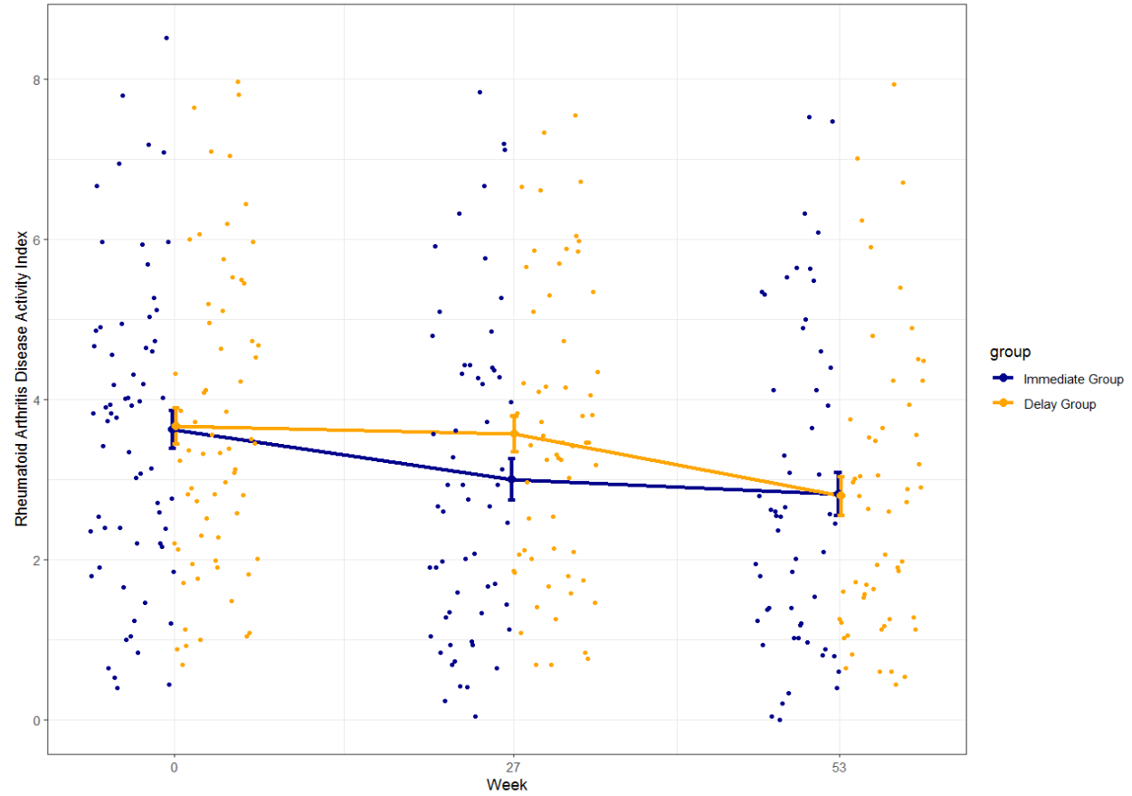


**Figure S1-1: Rheumatoid Arthritis Disease Activity Index (RADAI)**


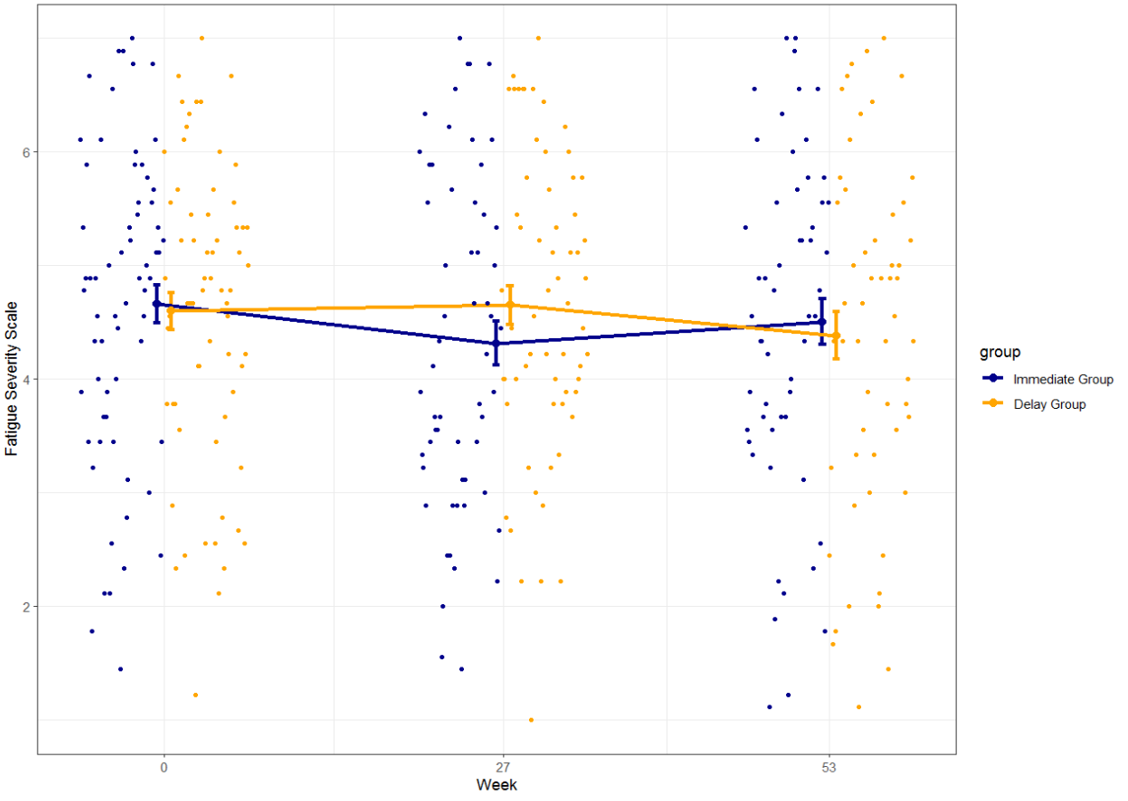


**Figure S1-2: Fatigue Severity Scale**


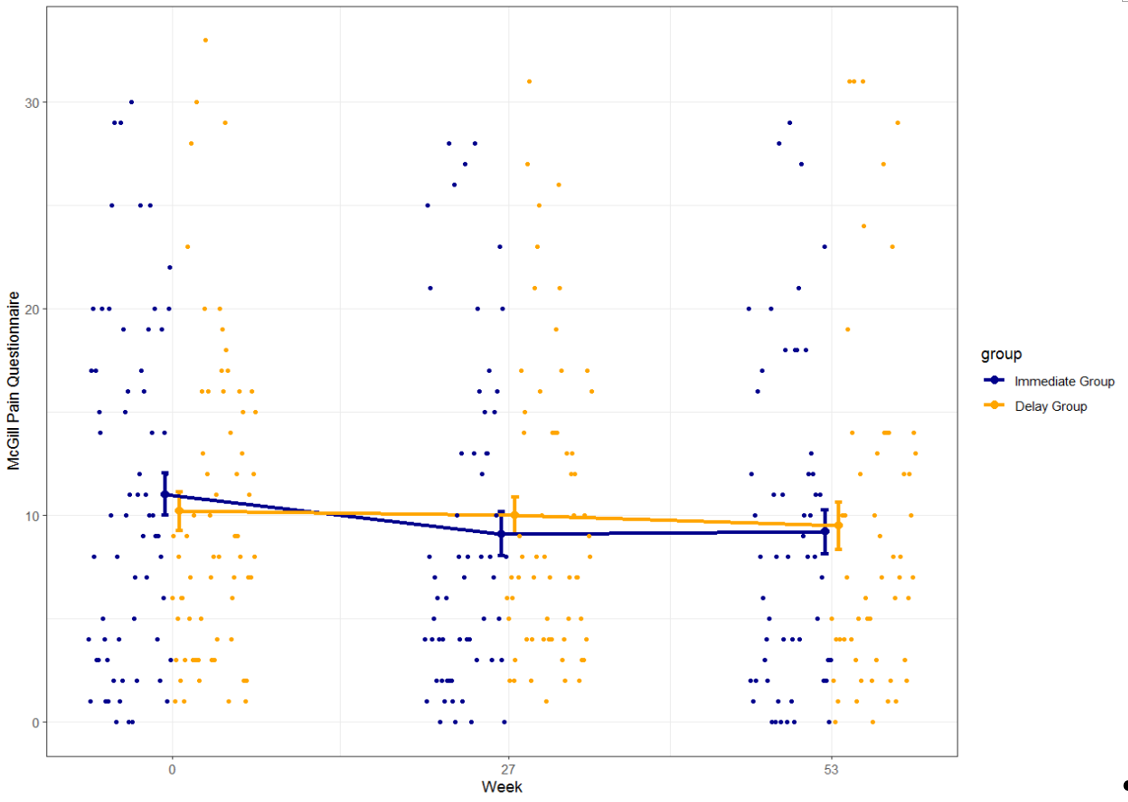


**Figure S1-3: McGill Pain Questionnaire**


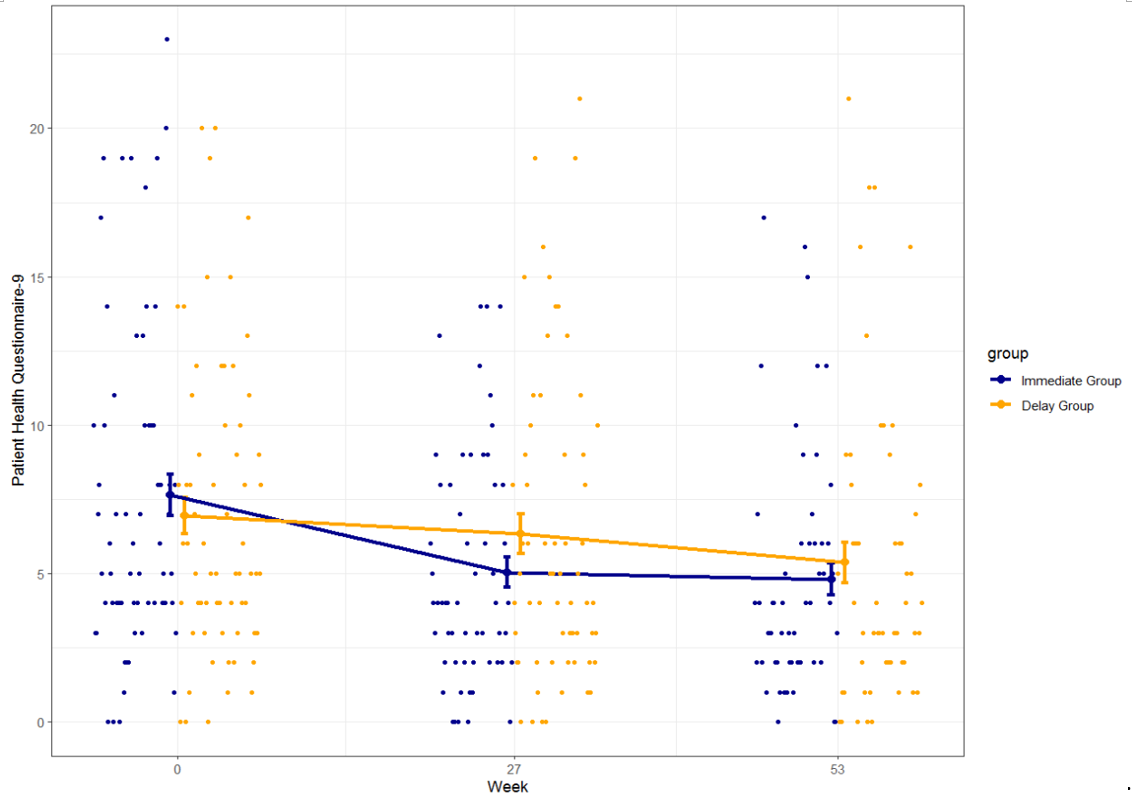


**Figure S1-4: Patient Health Questionnaire (PHQ-9)**
